# Novel insights into the ontogeny of basophils

**DOI:** 10.3389/falgy.2024.1402841

**Published:** 2024-05-13

**Authors:** Kensuke Miyake, Junya Ito, Hajime Karasuyama

**Affiliations:** Institute of Research, Tokyo Medical and Dental University (TMDU), Tokyo, Japan

**Keywords:** single-cell transcriptome, basophil, eosinophil, erythrocyte, megakaryocyte, progenitor, pre-basophil

## Abstract

Basophils are the least common granulocytes, accounting for <1% of peripheral blood leukocytes. In the last 20 years, analytical tools for mouse basophils have been developed, and we now recognize that basophils play critical roles in various immune reactions, including the development of allergic inflammation and protective immunity against parasites. Moreover, the combined use of flow cytometric analyses and knockout mice has uncovered several progenitor cells committed to basophils in mice. Recently, advancements in single-cell RNA sequencing (scRNA-seq) technologies have challenged the classical view of the differentiation of various hematopoietic cell lineages. This is also true for basophil differentiation, and studies using scRNA-seq analysis have provided novel insights into basophil differentiation, including the association of basophil differentiation with that of erythrocyte/megakaryocyte and the discovery of novel basophil progenitor cells in the mouse bone marrow. In this review, we summarize the recent findings of basophil ontogeny in both mice and humans, mainly focusing on studies using scRNA-seq analyses.

## 1 Introduction

Basophils are the rarest granulocytes, accounting for ∼1% of peripheral blood leukocytes in both mice and humans. Since their discovery more than 140 years ago by Paul Ehrlich, the functional significance of basophils in health and diseases remained largely unnoticed, partly due to their rarity and functional similarities with tissue-resident mast cells, including basophilic granules, surface expression of high-affinity IgE receptors (FcεRI), and release of histamines upon activation. Hence, basophils had erroneously been considered as blood-circulating mast cells that exert only redundant functions to mast cells. However, recent studies have revealed that basophils and mast cells display distinct transcriptomic signatures (1), indicating the non-redundant role of basophils. Intriguingly, basophils are found in the peripheral blood of some snapping turtles and teleost fishes (2, 3), indicating the evolutional conservation of basophils and the unique role of basophils in animals.

In these 10–20 years, researchers have developed an array of analytical tools for mouse basophils, such as basophil-depleting antibodies, engineered mice that can specifically deplete basophils, basophil reporter mice, and basophil-specific Cre-expressing mice (Table 1) (4–16). Under such tools, basophils are now found to play a critical role in various immune reactions, including chronic allergic inflammation, protective immunity against parasites, autoimmune reactions, and tumor immunity (17–20). Recent advancements in single-cell transcriptomic techniques have shed new light on the understanding of basophil biology. Studies using single-cell RNA sequencing (scRNA-seq) analysis have elucidated the critical roles of basophils in alveolar macrophage maturation, repair from myocardial infarction, kidney fibrosis, and induction and resolution of skin allergic inflammation (21–26). Moreover, single-cell transcriptomics have also revealed the differentiation trajectory of basophils and mast cells. Fate tracking experiments and single-cell transcriptomic analyses have identified the developmental origins of mast cells which is discussed in detail by other reviews (27–29). Therefore, in this review, we will focus on novel insights into the ontogeny of basophils, mainly identified by using single-cell transcriptomic techniques.

**Table 1:** Analytical tools for mouse basophil research.

Names	Description	References
(1) Basophil-depletion antibody		
Ba103 antibody (Ba160 antibody)	Rat monoclonal antibody against mouse CD200R3 specifically expressed on the surface of basophils and mast cells.	(4)
MAR-1 antibody	Hamster monoclonal antibody against mouse FcεRIα expressed on basophils, mast cells, and some subset of inflammatory dendritic cells.	(5)
(2) Basophil-depletion mice and basophil-deficient mice		
*Mcpt8*^DTR^ mice	Knock-in mice wherein human diphtheria toxin receptor (DTR) gene is inserted downstream of *Mcpt8* gene specifically expressed in basophils.	(6)
Bas-TRECK mice	Transgenic mice expressing human DTR gene under the control of 3′ UTR element of *Il4* gene locus selectively utilized by basophils.	(7)
BasoDTR mice	Transgenic mice expressing human DTR gene under the control of the promotor region of mouse *Enpp3* (encoding CD203c) gene.	(8)
*Mcpt8*Cre mice	Transgenic mice expressing Cre gene under the control of the promotor region of the *Mcpt8* gene. These mice lack basophils from birth possibly due to Cre toxicity.	(9)
(3) Basophil-specific Cre-expressing mice		
Basoph8 mice	Knock-in mice wherein eYFP-IRES-Cre cassette is inserted downstream of exon 5 of the *Mcpt8* gene. These mice can also be used as YFP-reporter mice. By crossbreeding with Cre-inducible DTR-expressing mice, these mice can be used as basophil-depletion mice.	(10, 11)
*Mcpt8*^iCre^ mice	Knock-in mice wherein exon 1 of *Mcpt8* gene is replaced by improved Cre (iCre) gene.	(12)
*Mcpt8*^iCreERT2^ mice	Transgenic mice expressing iCre-ERT2 cassette under the control of the *Mcpt8* promotor. These mice are tamoxifen-inducible Cre-expressing mice specifically in basophils.	(13)
CT-M8 mice	Knock-in mice wherein IRES-Cre-T2A-tdTomato cassette is inserted downstream of exon 5 of *Mcpt8* gene.	(14)
(4) Basophil reporter mice		
*Mcpt8*^GFP^ mice	Transgenic mice expressing enhanced GFP (eGFP) gene under the control of the *Mcpt8* promotor.	(15)
*Myb*-68 GFP mice	Transgenic mice expressing eGFP gene under the control of the *Myb-68* enhancer*.*	(16)

## 2 Progenitor populations committed to basophils and mast cells

Differentiation and maturation of basophils occur in the bone marrow, and mature basophils circulate in the bloodstream under homeostatic conditions (30, 31). In contrast, mucosal-type mast cells initially differentiate within the bone marrow, and immature mast cells migrate to the tissues where they undergo further maturation (27–29). Moreover, recent fate tracking studies identified that connective tissue-type mast cells arise from embryonic origin and appear to be independent of bone marrow progenitor under homeostatic conditions (32–34). Several progenitor populations which possess potentials to differentiate into basophils or mast cells are identified in mouse bone marrow (Table 2) (35–42). The unipotent basophil progenitors (BaPs) and progenitors for mast cells (mast cell progenitors; MCPs) are identified in the mouse bone marrow (35) and in the intestine, spleen and bone marrow (35–37), respectively.

**Table 2:** Progenitor cells committed to basophils or mast cells.

Names	Description	Tissue	References
(1) Bipotential progenitor for basophils and mast cells			
Pre-BMP	Lin^−^ Sca-1^−^ cKit^+^ CD34^+^ FcγRII/III^hi^ FcεRIα^+^	Bone marrow	(38)
Pro-BMP	Lin^−^ Sca-1^−^ cKit^+^ CD34^+^ FcγRII/III^hi^ E-cadherin^hi^ FcεRIα^+^	Bone marrow	(39)
BMCP	Lin^−^ cKit^+^ CD34^+^ FcγRII/III^hi^ Integrin β7^hi^	Spleen	(35)
		Bone marrow	(40)
(2) Basophil-committed progenitors			
BaP	Lin^−^ cKit^−^ CD34^+^ FcεRIα^+^	Bone marrow	(35)
Prebasophil	CD200R3^+^ cKit^−^CLEC12A^hi^ (FcεRIα^hi^)	Bone marrow	(41)
tBaso	Lin^−^ cKit^−^CD34^−^CD200R3^+^ FcεRIα^hi^	Bone marrow	(42)
(3) Mast cell-committed progenitors			
MCP	Lin^−^ Sca-1^−^ cKit^+^ Ly6C^−^ FcεRIα^−^ CD27^−^ Integrin β7^hi^ IL-33R^+^	Bone marrow	(36)
		Spleen	(37)
	Lin^−^ CD45^+^ FcεRIα^lo^ CD34^+^ Integrin β7^+^	Small intestine	(35)

In mice, the differentiation trajectory of basophils and mast cells are closely overlapped. Consistent with this assumption, *ex vivo* culture of bone marrow-derived progenitor cells can generate both basophils and mast cells, indicating the presence of common basophil and mast cell precursors in the bone marrow (43). Indeed, bipotential progenitor cells that can produce both basophils and mast cells have been identified in the mouse bone marrow and spleen. In 2013, Qi et al. identified that FcεRIα^hi^ granulocyte–macrophage progenitors (GMPs) in the bone marrow possess a highly enriched capacity for differentiating into basophils and mast cells, and FcεRIα^hi^ GMPs were considered pre-basophil/mast cell progenitors (pre-BMPs) (38). Single-cell colony-forming assay identified that ∼40% of pre-BMPs can produce both basophil and mast cell lineages, indicating the presence of basophil–mast cell common progenitors in pre-BMP populations. A later study has identified E-cadherin^+^FcεRIα^−^ GMPs named as pro-BMPs in the bone marrow, which are the immediate precursor of pre-BMPs and can differentiate into basophils and mast cells (39).

Besides pre- and pro-BMPs in the bone marrow, bipotential basophil/mast cell progenitor cells defined as Lin^−^Sca-1^−^c-Kit^+^ Integrin β7^hi^ FcγRII/III^hi^ basophil/mast cell progenitors (BMCPs) are also found in the mouse spleen (35). Colony-forming experiments identified that BMCPs can differentiate into both basophils and mast cells. However, the differentiation potential of spleen BMCPs is controversial because a later study indicates that BMCPs in the spleen preferentially differentiate into mast cells and have little capacity for differentiating into basophils (44). Although the first report only identified BMCPs in the spleen and not in the bone marrow (35), a recent study has identified the presence of BMCP in the mouse bone marrow (40). Single-cell colony-forming assay revealed that ∼10% of BMCPs in the bone marrow can differentiate into both basophils and mast cells. Collectively, in mice, bipotential basophil and mast cell progenitor cells, including pre-BMPs and BMCPs, produce unipotent progenitor cells of each cell type, such as BaPs and MCPs, which further differentiate into mature basophils and mast cells, respectively. However, whether BMCPs and pre-BMPs are the different cell types is unclear. Comprehensive single-cell transcriptomic analysis that can characterize both BMCPs and pre-BMPs is required to resolve this problem.

Human basophils have been believed to differentiate from common basophil/eosinophil progenitors (45). In the 1980s, the methylcellulose-based culture of human bone marrow or peripheral blood cells was reported to promote the generation of hybrid cells containing both basophilic and eosinophilic granules (45, 46). Moreover, hybrid basophilic/eosinophilic cells can also be detected in the peripheral blood or bone marrow in patients with myeloid leukemia (47). Another study showed that hybrid basophilic/eosinophilic cells can be generated *in vitro* from CD133^lo/−^CD34^+^ cells derived from umbilical cord blood (48). Therefore, basophils may differentiate from bipotential basophil/eosinophil progenitors in humans, although a later study identified that hybrid basophilic/eosinophilic cells mainly differentiate into eosinophils but not into basophils (49). However, whether the differentiation of human basophils is associated with that of human mast cells is unclear, in contrast to the case of mouse basophils.

## 3 Basophil differentiation trajectory uncovered with scRNA-seq analyses

**Figure 1:**
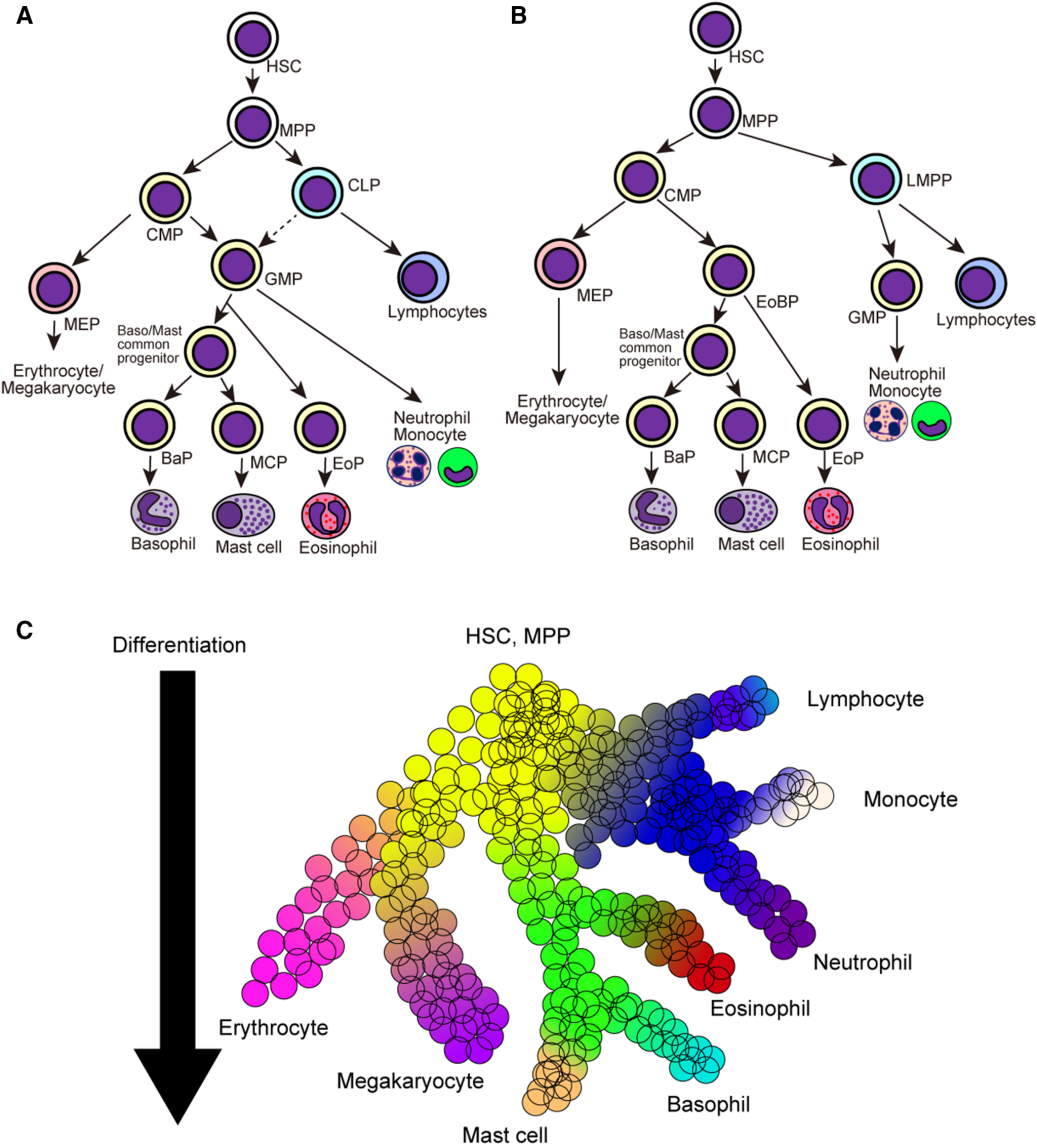
Classical and revised model of hematopoietic cell differentiation. (**A**) Classical model of hematopoietic cell differentiation tree. Hematopoietic stem cells (HSCs) and multi-potential progenitors (MPPs) firstly differentiate into common myeloid precursors (CMPs) and common lymphoid precursors (CLPs), and CMPs further differentiate into megakaryocyte/erythrocyte precursors (MEPs) and granulocyte/macrophage precursors (GMPs). (**B**) The revised model of hematopoietic cell differentiation. HSCs and MPPs differentiate into common myeloid precursors (CMPs) and lympho-myeloid primed progenitors (LMPPs). CMPs differentiate into MEPs and eosinophil-basophil progenitor (EoBP), while LMPPs differentiate into GMPs and lymphoid progenitor cells. (**C**) Continuous hematopoietic differentiation landscape model emerged after the advancements in the scRNA-seq analysis. Hematopoietic cell lineages continuously change their cellular states during the differentiation. HSC, hematopoietic stem cell; MPP, multi-potential progenitor; CMP, common myeloid progenitor; CLP, common lymphoid progenitor; LMPP, lympho-myeloid primed progenitor; MEP, megakaryocyte/erythrocyte progenitor; GMP, granulocyte/macrophage progenitor; BaP, basophil progenitor; MCP, mast cell progenitor; EoP, eosinophil progenitor; EoBP, eosinophil/basophil progenitor.

Classically, hematopoietic stem cells initially segregate into common myeloid progenitors (CMPs) and common lymphoid progenitors (CLPs), and CMPs further differentiate into megakaryocyte/erythrocyte progenitors (MEPs) and granulocyte/macrophage progenitors (GMPs) (Figure 1A) (50–52). In this model, all members of granulocytes, namely basophils, eosinophils, and neutrophils, are derived from the same precursor cells. Providing that basophils and mast cells differentiate from bipotential common progenitor cells in mice, basophils and mast cells may be derived from GMPs. In line with this assumption, both pre-BMPs and BMCPs can be induced *ex vivo* from GMPs (35, 38). However, several studies have challenged the classical model of hematopoietic cell differentiation. Franco et al. identified that single-cell culture of Sca-1^lo^Flt3^−^ CMPs but not Sca-1^lo^ GMPs can produce mast cells, indicating the differentiation trajectory of mast cells and granulocytes can be segregated at the CMP stage (53). Moreover, recent studies using scRNA-seq have identified that the differentiation trajectory of basophil/eosinophil/mast cell lineages is coupled with that of erythrocyte/megakaryocyte rather than that of neutrophil/monocyte in both mice and humans (54–58), leading to the generation of the revised model of hematopoietic cell differentiation (Figure 1B).

Advancements in single-cell transcriptomic techniques have exposed novel and comprehensive insights into the differentiation trajectories of various hematopoietic cell lineages. A series of single-cell transcriptomic datasets have challenged the classical stepwise hematopoietic cell differentiation model wherein cells are transitioned to the discrete progenitor states during their differentiation. Instead, multiple studies have proposed the continuous differentiation landscape model, which indicates a continuous change in cellular states during the differentiation of various hematopoietic cell lineages (Figure 1C) (59–61). This is also true for basophil/mast cell differentiation, and recent studies with scRNA-seq analysis have elucidated continuous gene expression changes during basophil differentiation (Table 3) (16, 40, 41, 54–58, 62–64). In the following sections, we summarize the recent findings in basophil differentiation mainly focusing on the following points: (1) the association of the basophil differentiation trajectory to the erythrocyte/megakaryocyte differentiation trajectory in mice and humans; (2) the association of basophil differentiation with the differentiation of mast cells or eosinophils; (3) the characterization of basophil progenitor cells located upstream of mature basophils.

**Table 3:** Single-cell transcriptomic datasets for the analysis of basophil/mast cell ontogeny.

Article	Cells analyzed in scRNA-seq	Platform
Mouse		
Tusi et al. (54)	cKit^+^ cells in the adult BM	inDrops
Dahlin et al. (40)	Lin^−^Sca-1^+^ cKit^+^ cells and Lin^−^ cKit^+^ cells in the WT BM	10X Genomics platform
Weinreb et al. (55)	Lin^−^Sca-1^+^ cKit^+^ cells and Lin^−^ cKit^+^ cells cultured *ex vivo* (lineage tracing)	inDrops
	Lin^−^Sca-1^+^ cKit^+^ cells and Lin^−^ cKit^+^ cells transferred to WT mice (lineage tracing)	
Drissen et al. (62)	pre-GM population in the BM	Fluidigm C1 platform
Dahlin et al. (40)	BMCPs and GMPs isolated from the BM	Samart-Seq2
Hamey et al. (63)	CD34^+^ BaPs and CD34^−^ basophils in the BM	Samart-Seq2
	P1 mast cell progenitor and mature mast cells in the peritoneum	
Miyake et al. (41)	bone marrow-derived basophils elicited from the WT BM	BD Rhapsody platform (TAS-seq method)
	*Mcpt8*-GFP^+^ basophils in the BM and spleen	
	CD200R3^+^ cKit^−^ basophils in the BM, spleen, and skin infection site after the second Nb infection	
Matsumura et al. (16)	*Myb*-68 GFP^+^ cells in the BM	10X Genomics platform
	*Myb*-68 GFP^+^ Ly6C^−^ GMPs cultured *ex vivo*	
Human		
Velten et al. (56)	Lin^−^ CD34^+^ CD38^−^ cells and Lin^−^ CD34^+^ CD38^+^ cells from the adult human bone marrow cells	QUARTZ-seq/modified Smart-seq2
Zheng et al. (57)	CD34^+^ cells from umbilical cord blood cells	Drop-seq
Pellin et al. (58)	Lin^−^ CD34^+^ CD164^+^/Lin^−^CD34^lo^CD164^hi^/Lin^−^CD34^−^CD164^hi^/Lin^−^CD34^−^CD164^lo^ isolated from the adult human bone marrow cells	inDrops
Drissen et al. (64)	CMP/MEP/GMP isolated from the adult human BM	Smart-seq2

### 3.1 Association of basophil differentiation trajectory to erythrocyte/megakaryocyte differentiation trajectory

Drissen et al. conducted a scRNA-seq analysis of mouse pre-granulocyte–macrophage progenitors (pre-GMs) and identified the presence of distinct pre-GM populations, namely GATA1^+^ and GATA1^−^ pre-GMs (62). *In vitro* culture experiments identified that GATA1^−^ pre-GMs preferentially differentiate into neutrophils and monocytes, whereas GATA1^+^ pre-GMs can differentiate into both eosinophil/mast cell and erythrocyte/megakaryocyte lineages. Tusi et al. conducted a scRNA-seq analysis of c-Kit^+^ cell populations isolated from adult mouse bone marrow by using the inDrops technique (54). Population balance analysis predicted that the differentiation trajectory of basophils/mast cells was coupled with erythrocyte differentiation rather than neutrophil or monocyte differentiation. Supporting this notion, ∼25% of single-cell cultured c-Kit^+^CD55^+^CD105^+^CD71^−^CD150^+^ progenitor cells produced both TER-119^+^ erythroid cells and FcεRI^+^ basophils. Weinreb et al. traced hematopoietic lineage differentiation by combining DNA barcoding with scRNA-seq technologies (55). They conducted scRNA-seq analysis of DNA-barcoded mouse hematopoietic stem cells cultured *ex vivo* for 2, 4, and 6 days. Lineage tracing identified the presence of progenitor cells that differentiate into basophil/mast cell/eosinophil and erythrocyte/megakaryocyte lineages. Lineage hierarchy tree constructed by clonal couplings inferred that the differentiation trajectory of basophil/mast cell/eosinophil is coupled with that of erythrocyte/megakaryocyte *in vitro*. However, the conclusion was different when the authors used scRNA-seq datasets obtained from *in vivo* differentiated DNA-barcoded hematopoietic stem cells, indicating further experimental evidence to verify the coupling of differentiation pathways between basophil/mast cell and erythrocyte lineages.

The association of basophil and erythrocyte differentiation is also reported in humans. Görgens et al. identified that CD133^lo/−^CD34^+^ hematopoietic progenitor cells produce both basophils/eosinophils and erythrocytes/megakaryocytes, although these progenitor populations have limited potential to differentiate into neutrophils (48). Drissen et al. conducted scRNA-seq analysis of Lin^−^CD34^+^CD38^+^CD123^+^CD45RA^−^ CMPs in the human bone marrow and identified the heterogeneity within the CMP population (64). Thus, they showed that CMP populations can be subdivided by surface expressions of two markers: CD114 (G-CSFR) and CD131 (IL-3Rβ). Single-cell culture experiments showed that CD114^+^ CMPs produce neutrophil and monocyte lineages, whereas CD131^+^ CMPs produce both eosinophil/mast cell/basophil and megakaryocyte/erythrocyte lineages.

A series of scRNA-seq analyses further support the association of basophil/eosinophil differentiation and erythrocyte/megakaryocyte differentiation. Velten et al. combined flow cytometry and scRNA-seq analyses of human Lin^−^CD34^+^ adult human bone marrow cells and identified that hematopoietic stem cells gradually acquire lineage-committed gene expression profiles during their differentiation (56). Based on the gene expression profiles, early progenitors committed to eosinophils/basophils/mast cells expressing *CLC*, *HDC*, and *PRG2* are identified in the Lin^−^CD34^+^CD38^+^ population. Progenitors of eosinophils/basophils/mast cells display CD34^+^CD38^+^CD10^−^CD45RA^−^CD135^mid^ MEP-like surface expression phenotype, indicating the close relationship between eosinophil/basophil/mast cell and erythrocyte/megakaryocyte differentiation. Similarly, scRNA-seq analysis of CD34^+^ human cord blood-derived progenitor cells revealed the coupling of eosinophil/basophil/mast cell differentiation to erythrocyte differentiation by using a minimum spanning tree algorithm (57). Pellin et al. conducted scRNA-seq analysis of Lin^−^ adult human bone marrow cells and showed the potential association between the differentiation trajectory of basophils and erythrocytes/megakaryocytes by using population balance analysis (58). *Ex vivo* culture experiments identified that Lin^−^CD34^+^CD135 (Flt3)^+^ bone marrow precursor cells preferentially differentiate into monocytes, whereas Lin^−^CD34^+^CD135^−^ cells can produce both basophils and megakaryocytes. A recent study identified that FcεRIα^+^Integrin-β7^+^CD203c^−^ CMPs can produce basophils, mast cells, and erythrocytes, whereas FcεRIα^+^CD203c^+^ CMPs produce only basophils and mast cells but not erythrocytes (65). These results support the notion that the differentiation trajectory of basophils/eosinophils/mast cells is associated with that of erythrocytes/megakaryocytes but not that of neutrophils/monocytes.

### 3.2 Association of the differentiation trajectory of basophils with that of mast cells and eosinophils

Besides the association of the differentiation trajectory of basophils with that of the erythrocytes/megakaryocytes, scRNA-seq analysis revealed the coupled differentiation pathways of basophils and mast cells in mice. Dahlin et al. confirmed the association between basophil and mast cell differentiation pathway by using scRNA-seq analysis of mouse hematopoietic progenitor populations in the bone marrow (40). Force-directed graph visualization identified that entry points into basophil and mast cell differentiation are close, indicating the coupled differentiation trajectory of basophils and mast cells. By contrast, the association of the basophil differentiation trajectory with that of eosinophils is less clarified in mice. The scRNA-seq datasets by Dahlin et al. mapped the eosinophil differentiation trajectory close to that of neutrophils (40). By contrast, scRNA-seq datasets of c-Kit^+^ bone marrow progenitor cells mapped basophils and eosinophils in the same cluster (54, 58), indicating the closely coupled differentiation trajectory. Assessment of the transcriptome of eosinophils by using single-cell RNA-seq is technically challenging, possibly because of the abundant amounts of RNase in their granules, their sensitivity to the shear stress, and degranulation. Recently, Gurtner et al. reported the scRNA-seq data of eosinophils isolated from various tissues in mice by preventing shear stress and resulting mRNA degradation (66). By using such approaches, the relationship among the ontogeny of eosinophil, mast cell, and basophil lineages would be clarified.

Contrary to the case of mice, the association of the basophil-differentiation trajectory with that of mast cells or eosinophils is less clear in humans. Because studies using scRNA-seq have positioned basophils close to mast cells or eosinophils (56, 57), the differentiation of basophils, mast cells, and eosinophils may be closely coupled to each other in humans.

### 3.3 Identification of basophil lineage-committed progenitors by using scRNA-seq

**Figure 2:**
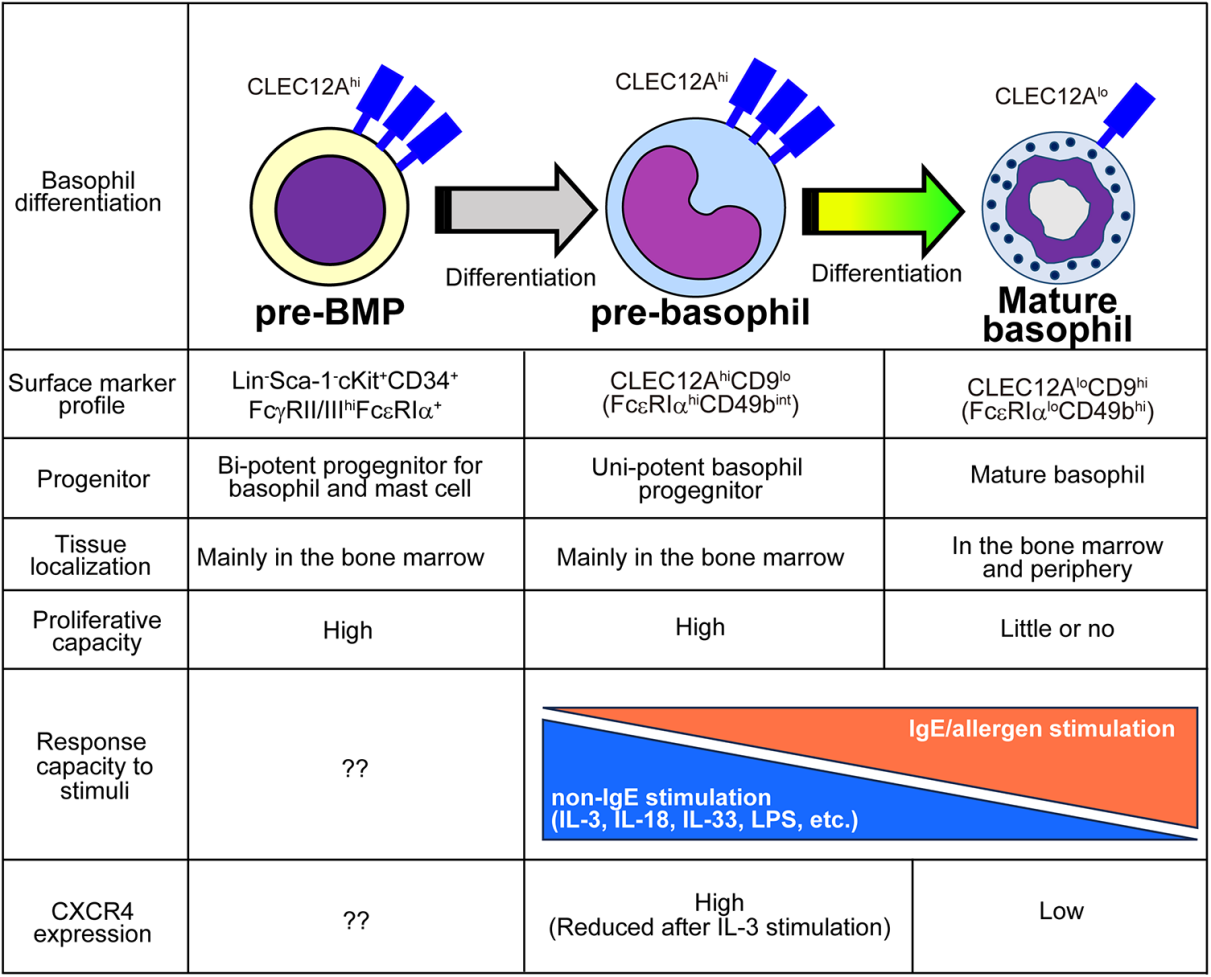
Characteristics of pre-BMPs, pre-basophils, and mature basophils. The distinct properties among pre-BMPs, pre-basophils and mature basophils are summarized.

In addition to the characterization of the basophil differentiation trajectory, single-cell transcriptomic analyses were also used to identify basophil-committed progenitor cell populations. By combining highly sensitive scRNA-seq (67) and flow cytometric analyses, Miyake et al. identified CLEC12A^hi^c-Kit^lo^ pre-basophils within the mouse bone marrow and bone marrow-derived basophils (BMBAs) (Figure 2) (41). CLEC12A^hi^ pre-basophils and CLEC12A^lo^ mature basophils showed distinct cell morphology and cell surface markers. CLEC12A^hi^ pre-basophils showed kidney-shaped indented nuclei with large cell bodies, whereas CLEC12A^lo^ mature basophils showed ring-shaped nuclei with smaller cell bodies. CLEC12A^hi^ pre-basophils and CLEC12A^lo^ mature basophils in the bone marrow showed FcεRIα^hi^CD49b^int^ and FcεRIα^lo^CD49b^hi^ surface expression profiles, respectively. RNA velocity and pseudotime trajectory analyses inferred the sequential differentiation trajectory from *Fcer1a*^hi^*Cd34*^hi^*Kit*^hi^ pre-BMP-like populations into *Clec12a*^hi^*Cd9*^int^*Kit*^lo^ pre-basophils that further differentiate into *Clec12a*^lo^*Cd9*^hi^*Kit*^lo^ mature basophils. Consistently, *ex vivo* culture of CLEC12A^hi^CD9^int^ pre-basophils for 24 h promoted the differentiation into CLEC12A^lo^CD9^hi^ mature basophils even without IL-3. Notably, CD34 ^+ ^FcεRIα BaPs also showed CLEC12A^hi^CD9^int^ phenotype, and only 10% of CLEC12A^hi^ populations showed positive but low expression of CD34, indicating the possibility that CD34^+^ BaPs are included in the CLEC12A^hi^ pre-basophil populations. Supporting this notion, scRNA-seq analysis revealed that a small fraction (10%–20%) of *Clec12a*^hi^ cells in the pre-basophil cluster showed *Cd34*^hi^*Kit*^lo^ phenotype. Transcriptomic analysis revealed that pre-basophils and mature basophils highly expressed genes associated with cell proliferation and effector functions, respectively. Consistently, pre-basophils showed higher proliferative capacity than mature basophils. By contrast, mature basophils showed greater capacity for degranulation and *de novo* cytokine production in response to antigen/IgE stimulation. Notably, when basophils are stimulated with non-IgE stimulation, such as IL-3, IL-3+ IL-18, IL-3 + IL-33, and IL-3 + LPS, pre-basophils produced a greater amount of IL-4 than mature basophils, indicating that the responsiveness to stimulants is different between pre-basophils and mature basophils. Similarly, CD34^+^ basophil progenitors produced a greater amount of type 2 and proinflammatory cytokines than mature basophils in response to IL-33 stimulation (68). Thus, the bulk transcriptomic analysis identified that pre-basophils showed limited gene expression changes in response to antigen/IgE stimulation although FcεRIα expression on pre-basophils was higher than mature basophils. Bulk transcriptomic analysis further showed that IL-3-stimulated pre-basophils and mature basophils showed distinct gene expression profiles.

Reanalysis of previously published scRNA-seq analysis dataset obtained by Weinreb et al. identified *Clec12a*^hi^ pre-basophils among *Mcpt8*^+^ basophil populations. Likewise, pre-basophil-like precursor populations were also reported by other groups. The scRNA-seq analysis of basophil lineage populations by using *Myb*-68 GFP^+^ cells identified two heterogeneous basophil populations, namely *Fcer1a*^hi^ basophil1 and *Fcer1a*^int^ basophil2 populations (16), which correspond to pre-basophils and mature basophils, respectively. Park et al. identified CD34^−^CD200R3^+^FcεRIα^hi^ transitional basophils (tBasos) in the bone marrow with high proliferative capacity (42).

Under homeostatic conditions, pre-basophils reside in the bone marrow and are rarely detected outside the bone marrow. By contrast, 7 days after infection with intestinal helminth *Nippostrongylus brasiliensis* (Nb), CLEC12A^hi^ pre-basophils egress the bone marrow and can be detected in the spleen, peripheral blood, and lungs. Notably, CLEC12A^hi^ pre-basophils detected in the infected lungs retained their proliferative capacity and expressed IL-4 comparable to mature basophils. Moreover, the scRNA-seq analysis of basophils accumulating in the skin infection site after the second Nb infection demonstrated that pre-basophils detected in the skin infection site show transcriptional profiles similar to those of pre-basophils in the bone marrow of noninfected mice.

IL-3 promotes blood basophilia during Nb infections (69, 70). Notably, mice treated with IL-3 complexes also showed the emergence of CLEC12A^hi^ pre-basophils in the peripheral blood. Therefore, IL-3 upregulation caused by helminth infection may promote the egress of pre-basophils from the bone marrow. Further mechanistic study identified that IL-3 promotes the downregulation of chemokine receptor CXCR4 on basophils in the bone marrow, which prevents the retention of pre-basophils in the bone marrow and promotes the appearance of pre-basophils in the periphery.

Contrary to the case of mouse basophils, the committed progenitors for human basophils remain less identified. Providing that human basophil-like cells can be induced by culturing CD34^+^ progenitor cells isolated from the adult human bone marrow in the presence of IL-3, progenitors possessing basophil-differentiation potential may be found in the CD34^+^ cells in the bone marrow (71). Similarly, human mast cells can be generated *ex vivo* by culturing CD34^+^c-Kit^+^ cells isolated from the peripheral blood (72, 73), indicating the presence of mast cell-committed progenitors in the peripheral blood CD34^+^c-Kit^+^ cells. In line with this, recent studies have discovered Lin^−^CD34^hi^c-Kit^int/hi^FcεRIα^+^ mast cell-committed precursor cells in the peripheral blood (74, 75).

## 4 Transcription factors regulating basophil/mast cell fates

**Figure 3:**
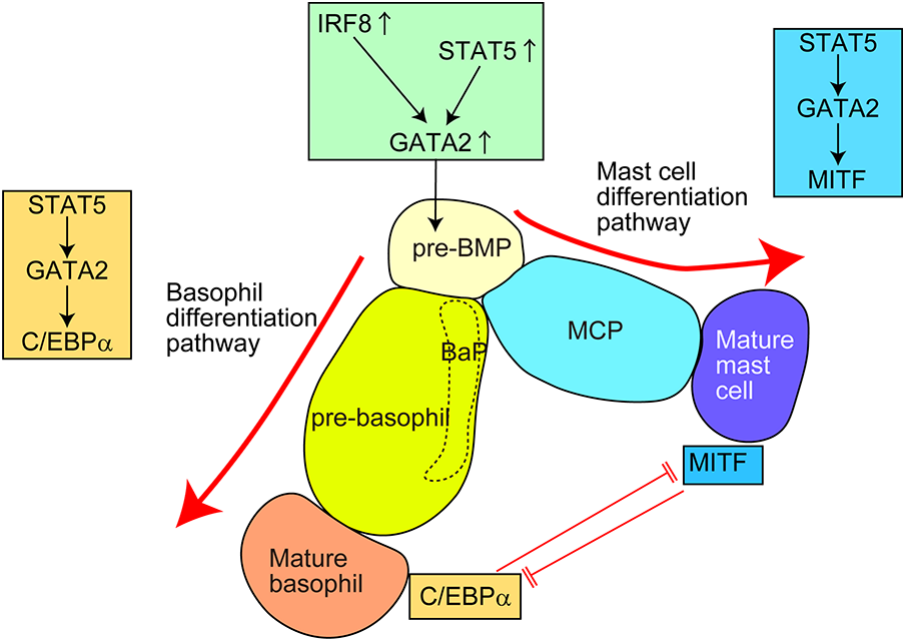
Transcription factors (TFs) regulating the differentiation of basophils and mast cells in mice. For the generation of common basophil/mast cell progenitors (pre-BMPs), the upregulation of GATA2, possibly caused by upregulation of STAT5 and/or IRF8, is required. For the differentiation into basophils, the STAT5–GATA2–C/EBPα signaling pathway is required. For the differentiation into mast cells, the STAT5–GATA2–MITF signaling pathway is required. Pre-BMP, pre-basophil/mast cell progenitor; BaP, basophil progenitor; MCP, mast cell progenitor.

Several transcription factors (TFs) regulate the differentiation into basophils and mast cells (Figure 3 and Table 4) (16, 37–39, 42, 44, 76–83). These TFs are divided into two groups: (1) TFs important for both basophil and mast cell lineages; (2) TFs important for basophils but not for mast cells, or vice versa.

**Table 4:** Transcription factors (TFs) regulating the fate of basophils and mast cells in mice.

Names	Mice/Intervention	Description	References
(1) TFs regulating both basophils and mast cells			
STAT5	*Stat5*^−*/*−^ (BM chimera)	Reduced number of BaPs, basophils (in the BM) and tissue mast cells	(76, 77)
	*Mx1*^Cre^ *Stat5a/b*^fl/−^	Moderate reduction of pre-BMPs, BaPs, and mature basophils in the BM	(38)
GATA2	*Rosa26*^CreER^ *Gata2*^fl/fl^	Reduced number basophils (in the BM) and mast cells (peritoneum, skin)	(78)
	*Rosa26*^CreER^ *Gata2*^fl/+^	Basophil number (in the BM): unchanged; reduced mast cells	
IRF8	*Irf8* ^−*/*−^	Reduced number of pre-BMPs, BaPs and basophils in the BM Tissue MCPs and mast cells: unchanged; reduced MCPs in the BM	(79)
GATA1	ΔdblGATA	Reduced number of BaPs, basophils (in the BM) Impaired responsiveness of basophils to antigen/IgE stimulation.	(80)
	GATA-1^low^ (neo*Δ*HS)	Increased number of basophils; reduced MCPs and tissue mast cells	(39)
*Myb*-68 enhancer	CRISPR deletion	Impaired generation of basophils and mast cells by *ex vivo* BM culture.	(16)
(2) Transcription factors regulating either basophils or mast cells			
C/EBPα	*Rosa26*^CreER^ *Cebpa*^fl/fl^	Reduced number of pre-BMPs in the BM Impaired generation of basophils from pre-BMPs	(38)
MITF	*Mitf* ^−/−^	Reduced mast cell number	(81, 82)
	*Mitf* ^Wsh/Wsh^	Impaired generation of mast cells from pre-BMPs	(38)
Ikaros	*Ikzf1* ^−/−^	Increased number of BaPs and mature basophils Reduced MCPs and mast cell number in the small intestine MCPs in the BM and skin mast cells are not affected	(37)
P1 RUNX1 promotor	*Runx1* ^P1N/P1N^	Reduced number of BaPs and mature basophils Tissue mast cell number is not affected	(44)
PLZF	*Zbtb16* ^−/−^	Reduced number of BaPs and mature basophils. Impaired responsiveness of basophils to antigen/IgE stimulation.	(83)
(3) Transcription factor regulating basophil function			
NFIL3	*Nfil3*ΔBaso (*Mcpt8*-Cre *Nfil3*^fl/fl^)	Number of tBaso and mature basophil: unchanged Impaired responsiveness of mature basophils to antigen/IgE stimulation	(42)

### 4.1 TFs regulating both basophil and mast cell fates

STAT5 and GATA2 regulate basophil and mast cell differentiation. Bone marrow chimeric mice reconstituted with STAT5-deficient cells showed a markedly reduced number of basophils and tissue mast cells (76, 77). Induced GATA2-deficiency significantly reduced the surface expression of FcεRIα and E-cadherin on basophils and c-Kit on mast cells (39, 78), which almost abolished the generation of basophils and tissue mast cells (38, 78). Considering that STAT5 can directly bind to the promotor region of GATA2 and GATA2 overexpression restores the phenotype of induced STAT5-deficiency, the STAT5–GATA2 signaling may be critical for basophil and mast cell differentiation. Moreover, the STAT5–GATA2 signaling is critical for the survival of mature basophils and mast cells. Notably, haploinsufficiency of *Gata2* gene affects the generation of mast cells but not basophils, indicating that mast cells are more heavily dependent on GATA2 TF than basophils, especially regarding their generation. A recent study further identified that GATA2 directly promoted chromatin remodeling at super-enhancer regions thus robustly regulating the expression of mast cell-associated genes and responsiveness to antigen/IgE stimuli (84). Thus, STAT5–GATA2 signaling is critical for both the differentiation and maintenance of basophils and mast cells in mice. However, the factor(s) inducing STAT5 activity for basophil/mast cell differentiation under homeostatic conditions remains unclear.

IL-3 is a growth factor promoting basophil differentiation and proliferation. The *ex vivo* culture of bone marrow cells in the presence of IL-3 induces the generation of BMBAs. Moreover, systemic administration of IL-3 complexes in mice increases the number of mature basophils and basophil precursor cells in the bone marrow and spleen (77, 85). Thymic stromal lymphopoietin (TSLP) is another growth factor that can promote the differentiation from basophil precursor cells into mature basophils. Systemic administration of TSLP increases the number of mature basophils in the spleen in an IL-3–IL-3R-independent manner (86). Notably, IL-3-elicited basophils and TSLP-elicited basophils show transcriptionally and functionally distinct properties. IL-3-elicited basophils are superior to TSLP-elicited basophils in the degranulation capacity in response to IgE-dependent stimuli. By contrast, TSLP-elicited basophils are more responsive to stimulation by IL-3, IL-18, and IL-33, and produce a greater amount of IL-4 compared with IL-3-elicited basophils. However, neither IL-3 nor TSLP is essential for basophil differentiation, which was evidenced by both IL-3 receptor-deficient mice and TSLP-receptor mice showing a normal number of basophils in the bone marrow (86). Moreover, basophil number was also intact even in the deficiency in the IL-3 and TSLP receptors (86), indicating that factor(s) other than IL-3 or TSLP played critical roles for the STAT5-mediated basophil differentiation.

IRF8 plays critical roles in the development of basophils and mast cells. Mechanistically, IRF8 regulates GATA2 expression in granulocyte progenitors; therefore, IRF8 deficiency impairs the generation of bipotent pre-BMPs in the bone marrow, leading to impaired development of basophils and mast cells in the bone marrow (79). IRF8-deficient mice showed significantly reduced pre-BMPs, BaPs, mature basophils, and MCPs in the bone marrow. However, IRF8-deficient mice showed a normal number of MCPs and mature mast cells in the tissues (e.g., intestine, skin, peritoneal cavity). Considering that the number of BMCPs in the spleen was unaffected by IRF8 deficiency, multiple pathways might have existed to retain tissue mast cell numbers.

The role of GATA1 is implicated in basophil and mast cell differentiation, although the contribution of GATA1 in mast cell differentiation remains controversial (87–89). Among hematopoietic cell lineages, GATA1 plays critical roles in the differentiation of erythrocytes, megakaryocytes, and eosinophils. GATA1-deficient mice result in embryonic lethality due to severe anemia, whereas ΔdblGATA mice lacking the double-GATA enhancer site in the *Gata1* promotor region show selective deficiency in eosinophil lineages with only mild anemia (90). The number of mature basophils and basophil progenitor cells in the bone marrow is partially reduced in ΔdblGATA mice, possibly due to the decreased *Gata1* expression in basophils (80). Moreover, basophils from ΔdblGATA mice show impaired degranulation and cytokine production in response to antigen/IgE stimulation. By contrast, GATA1-low mutant mice (91) that lack the upstream enhancer and promotor sequences of *Gata1* gene displayed an increased frequency of basophils in the bone marrow. Interestingly, basophils from GATA1-low mutant mice show reduced CD49b expression, indicating the possibility that GATA1 directly regulates CD49b expression or GATA1 promotes the differentiation from CD49b^lo^ pre-basophils into CD49b^hi^ mature basophils. Importantly, GATA1-low mutant mice showed increased *Gata2* expression in basophils, whereas ΔdblGATA mice showed normal *Gata2* expression in basophils (39, 80). Therefore, the discrepancy in the basophil numbers between ΔdblGATA mice and GATA1-low mutant mice might stem from the extent of the GATA2 expression in basophils.

A recent report identified that the use of the *Myb*-68kb enhancer region is largely restricted to basophil and mast cell lineages (16). CRISPR-mediated depletion of *Myb*-68 enhancer results in the reduced *Myb* expression and impaired generation of basophils and mast cells *in vitro*, indicating the role of *Myb*-68 enhancer in basophil and mast cell differentiation.

### 4.2 TFs regulating the fate of basophils but not mast cells

C/EBPα and MITF are critical TFs regulating basophil and mast cell differentiation, respectively (38). For differentiation into basophils, the STAT5–GATA2 signaling induces C/EBPα activity to promote basophil-associated gene expression. By contrast, for differentiation into mast cells, the STAT5 signaling induces MITF activity to promote mast cell-associated gene expression. In line with this, MITF-deficient mice lack mature mast cells in various tissues (81, 82). C/EBPα and MITF repress the expression of other genes by directly binding to the promotor region of MITF and CEBP/α gene, respectively. Consistently, inducible knockdown of the C/EBPα gene promotes the differentiation from pre-BMPs to mast cells. Interestingly, inducible loss of the C/EBPα gene in mature basophils induces the generation of c-Kit^+^ mast cell-like cells. Conversely, approximately half of the bone marrow cells from MITF-mutant mice failed to generate c-Kit^+^CD49b^+^ mast cells but generated c-Kit^−^CD49b^+^ basophil-like cells when cultured in the presence of IL-3 for 28 days. A similar phenomenon was observed in Ikaros-deficient mice (37). When bone marrow cells from Ikaros-deficient mice were cultured with IL-3 and stem cell factor (SCF) for 6 weeks, some cells showed c-Kit^−^FcεRIα^+^ basophil-like surface expression phenotype. Mechanistically, Ikaros directly binds to the promotor region of *Cebpa* and represses the C/EBPα expression possibly through histone modification.

RUNX1, a critical regulator of hematopoietic stem cells, is involved in basophil differentiation. Distal (P1) and proximal (P2) promotors regulate RUNX1 expression (92). Deficiency in P1 promotor results in significantly reduced mature basophils and basophil progenitor cells in the bone marrow (44). However, P1-RUNX1 deficiency has a limited impact on the number of mast cells and eosinophils, indicating the critical roles of P1-RUNX1 in basophil lineage differentiation. The zinc-finger TF promyelocytic leukemia zinc finger (PLZF encoded by the *Zbtb16* gene) is essential for the development of several innate lymphoid cells, including NKT cells, mucosal-associated invariant T (MAIT) cells, and group 2 innate lymphoid cells (ILC2s) (93). A recent study showed that *Zbtb16* was highly expressed in basophil progenitor cells (83). PLZF deficiency partially reduced the number of basophil progenitors and mature basophils in the bone marrow. PLZF deficiency also influenced IL-4 production of basophils in response to IgE-dependent stimulation. Therefore, PLZF plays key roles in the development and function of basophils. Although PLZF is expressed in mature mast cells, the effect of PLZF deficiency on mast cells remains unclear.

A recent report has identified that the TF NFIL3 is upregulated during basophil maturation. However, basophil-specific *Nifl3*-deficient mice show a normal number of mature basophil and basophil precursor (tBaso) populations, indicating the possible roles of NFIL3 in the effector functions of mature basophils. Indeed, basophil-specific *Nifl3*-deficiency partly impairs IgE-mediated activation of mature basophils (42). Moreover, basophil-specific *Nifl3*-deficient mice show reduced ear thickening in the hapten oxazolone-induced atopic dermatitis model (42), possibly due to the reduced IL-4 expression in skin-infiltrating basophils.

### 4.3 TFs involved in human basophil differentiation

A genetic association study using whole genome sequencing data from Estonian Biobank identified the strong association of basophil counts with the SNPs in *CEBPA* and *GATA2* gene loci (94), indicating that GATA2 and CEBP/α possibly regulate basophil differentiation in humans. The mutation in the +39-kb enhancer region of *CEBPA* (rs787444187) influences the basophil count but not the number of other myeloid lineages. Interestingly, ATAC-seq analysis identified that the open chromatin region containing *CEBPA* +39-kb enhancer is only present in CMPs but not in GMPs or MEPs, indicating that basophils possibly differentiate from CMPs but not from GMPs or MEPs in humans, which is consistent with the previous findings. When the *CEBPA* +39-kb enhancer region is mutated in human hematopoietic progenitor cells, the differentiation into basophils is partially blocked, but the differentiation into mast cells is rather enhanced (94), possibly through reduced *CEBPA* expression.

GATA2 deficiency syndrome is caused by heterozygous and loss-of-function mutation in GATA2 and displays severe abnormalities in multiple myeloid and lymphoid lineages, including monocytopenia, neutropenia, and dendritic cell deficiency (95). Moreover, patients with GATA2 deficiency syndrome develop myeloid neoplasms, including myelodysplastic syndrome and acute myeloid leukemia, with a median age of onset at 17 years. Consistent with the findings in *Gata2*-deficient mice, mast cells generated from hematopoietic progenitor cells isolated from GATA2 deficiency syndrome showed reduced FcεRIα and c-Kit expressions, resulting in the defective degranulation capacity in response to antigen/IgE and SCF stimulation (96). Moreover, patients with GATA2 deficiency syndrome have decreased surface expression of FcεRIα on peripheral blood basophils. Interestingly, patients with GATA2-deficiency syndrome had a reduced prevalence of IgE-medicated hypersensitivity syndrome possibly due to the reduced FcεRI expression on basophils and mast cells. However, whether GATA2 deficiency in humans affects the generation of basophils and mast cells in the bone marrow remains unclear, although the frequency of basophils in the peripheral blood is unaffected by GATA2 deficiency in humans.

## 5 Conclusion and perspectives

Recent advancements in the development of scRNA-seq techniques have brought us novel insights into the developmental pathways of hematopoietic cell lineages. This is also the case in the differentiation of basophils and mast cells, and recent studies using scRNA-seq analysis have elucidated the differentiation trajectory of basophils and mast cells in both mice and humans. Indeed, scRNA-seq analyses have confirmed the associated differentiation trajectory between basophils and mast cells in mice. Moreover, a series of scRNA-seq analyses have shown the potential coupling of basophil-differentiation pathway with the erythrocyte/megakaryocyte differentiation pathway in mice and humans. In addition, scRNA-seq studies have identified a novel progenitor population of basophils in the mouse bone marrow.

Knockout mice studies have elucidated several TFs regulating the fate of basophils and mast cells in mice. Further studies using both knockout mice and scRNA-seq analysis would further provide valuable insights into the mechanisms of basophil differentiation in mice. Compared with the case of mouse basophils, TFs regulating human basophils remain unclear. The combined use of scRNA-seq analysis with CRISPR screening or other perturbations (97, 98) would further accelerate our understanding of human basophil differentiation, which might connect to the identification of novel drug targets in basophil-related disorders, including allergic diseases and parasitic infections.
